# P-1072. Epidemiology of Stenotrophomonas maltophilia infections in adults hospitalized in a referral hospital in Nicaragua

**DOI:** 10.1093/ofid/ofaf695.1267

**Published:** 2026-01-11

**Authors:** Omar Gutiérrez-Zúniga, Kevin Gavarrete-Rivas, Sunaya Marenco-Avilés, Roger Maliaños-Miranda, Guillermo D Porras-Cortés

**Affiliations:** Hospital Dr. Fernando Vélez Paiz, Masaya, Masaya, Nicaragua; Hospital Dr. Fernando Vélez Paiz, Masaya, Masaya, Nicaragua; Hospital Dr. Fernando Vélez Paiz, Masaya, Masaya, Nicaragua; Hospital Dr. Fernando Vélez Paiz, Masaya, Masaya, Nicaragua; Hospital Dr. Fernando Vélez Paiz, Masaya, Masaya, Nicaragua

## Abstract

**Background:**

*Stenotrophomonas maltophilia* is a pathogen whose relevance is increasing both due to the number of cases and the emerging resistance pattern it has. The behavior of infections by this microorganism in each institution must be studied. The objective of this study was to analyze the epidemiological behavior of *S. maltophilia* infections in adults admitted to the Dr. Fernando Vélez Paiz Hospital, a referral center in Managua, Nicaragua. The resistance pattern is described and risk factors for mortality in patients with infection by this bacterium are identified.

**Figure 1.**
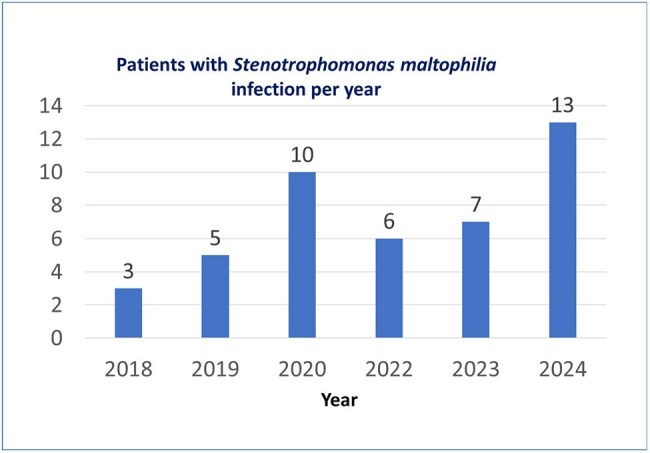
Number of patients with Stenotrophomonas maltophilia infection per year. Hospital Dr. Fernando Vélez Paiz. Managua, Nicaragua 2018-2024

**Table 1.**
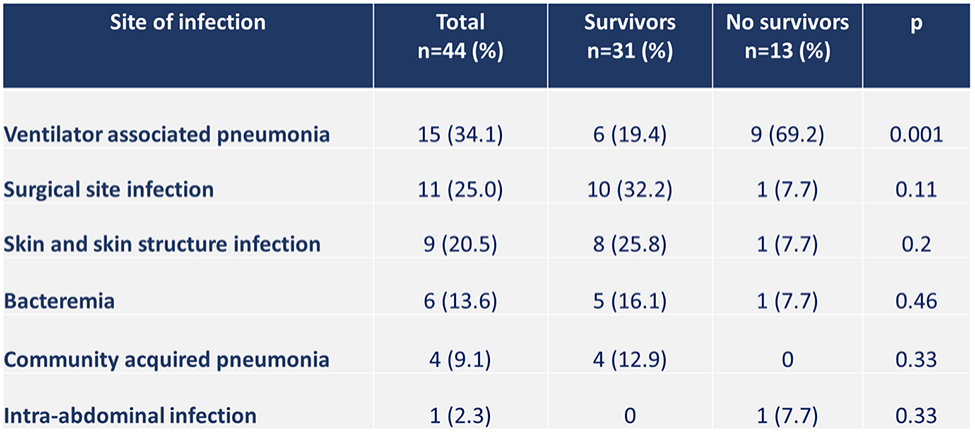
Site of infection caused by Stenotrophomonas maltophilia. Hospital Dr. Fernando Vélez Paiz. Managua, Nicaragua. 2018-2024.

**Methods:**

This is an observational, retrospective, cross-sectional study, conducted between April 2018 and December 2024. A total of 54 patients with documented *S. maltophilia* infection were identified but 10 of them were excluded from the analysis. Different variables including comorbidities, site and severity of the infection were evaluated. The proportions of the qualitative variables and the mean with standard deviation of the quantitative variables were established. When performing an analysis of risk factors for mortality, an analysis of odds ratios and 95% confidence interval were performed.

**Table 2.**
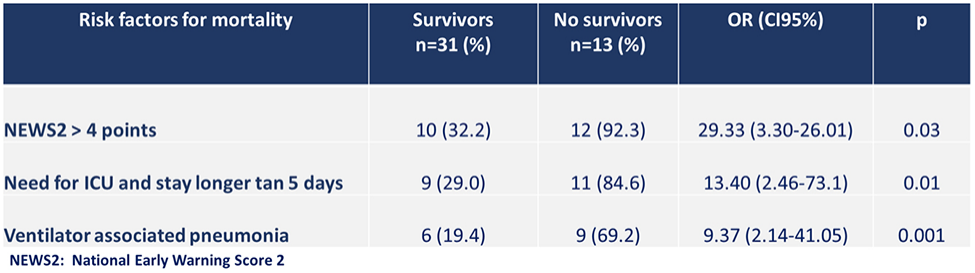
Risk factors for mortality in patients with Stenotrophomonas maltophilia infection. Hospital Dr. Fernando Vélez Paiz. Managua, Nicaragua. 2018-2024

**Table 3.**
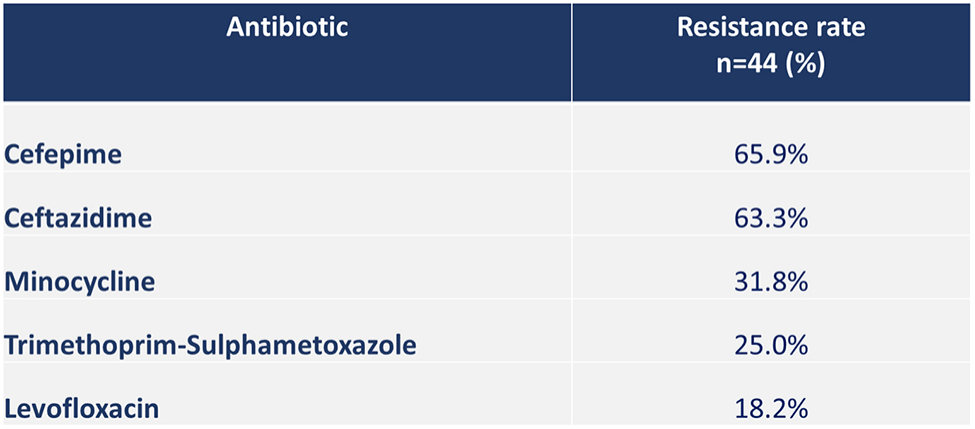
Resistance rate of Stenotrophomonas maltophilia to selected antibiotics. Hospital Dr. Fernando Vélez Paiz. 2018-2024

**Results:**

A total of 44 patients were studied, of which 13 died (29.5%). Cases of *S. maltophilia* infections increased from 3 in 2018 to 13 in 2024 (Figure 1). The mean age of the population was 53.1 ± 19.5 years old. The most frequent comorbidities were diabetes (43.2%) and hypertension (27.3%). The most common infection associated with *S. maltophilia* was ventilator-associated pneumonia (VAP) followed by surgical site infections and skin and skin structure infections (Table 1). VAP was more common in patients who died; the odds ratio (CI95%) for mortality in patients with VAP was 9.37 (2.14-41.05) (Table 2). Other risk factors for dying were a score greater than 4 on the NEWS2 score and need for ICU with a stay longer than 5 days. A high rate of resistance to ceftazidime was found (63.3%), and the rate of resistance to trimethoprim-sulfamethoxazole and levofloxacin was 25% and 18%, respectively (Table 3).

**Conclusion:**

Most *Stenotrophomonas maltophilia* infections are related to VAP. The factors associated with mortality were having greater than 4 points on the NEWS2 score, need for ICU with a stay longer than 5 days, and VAP.

**Disclosures:**

All Authors: No reported disclosures

